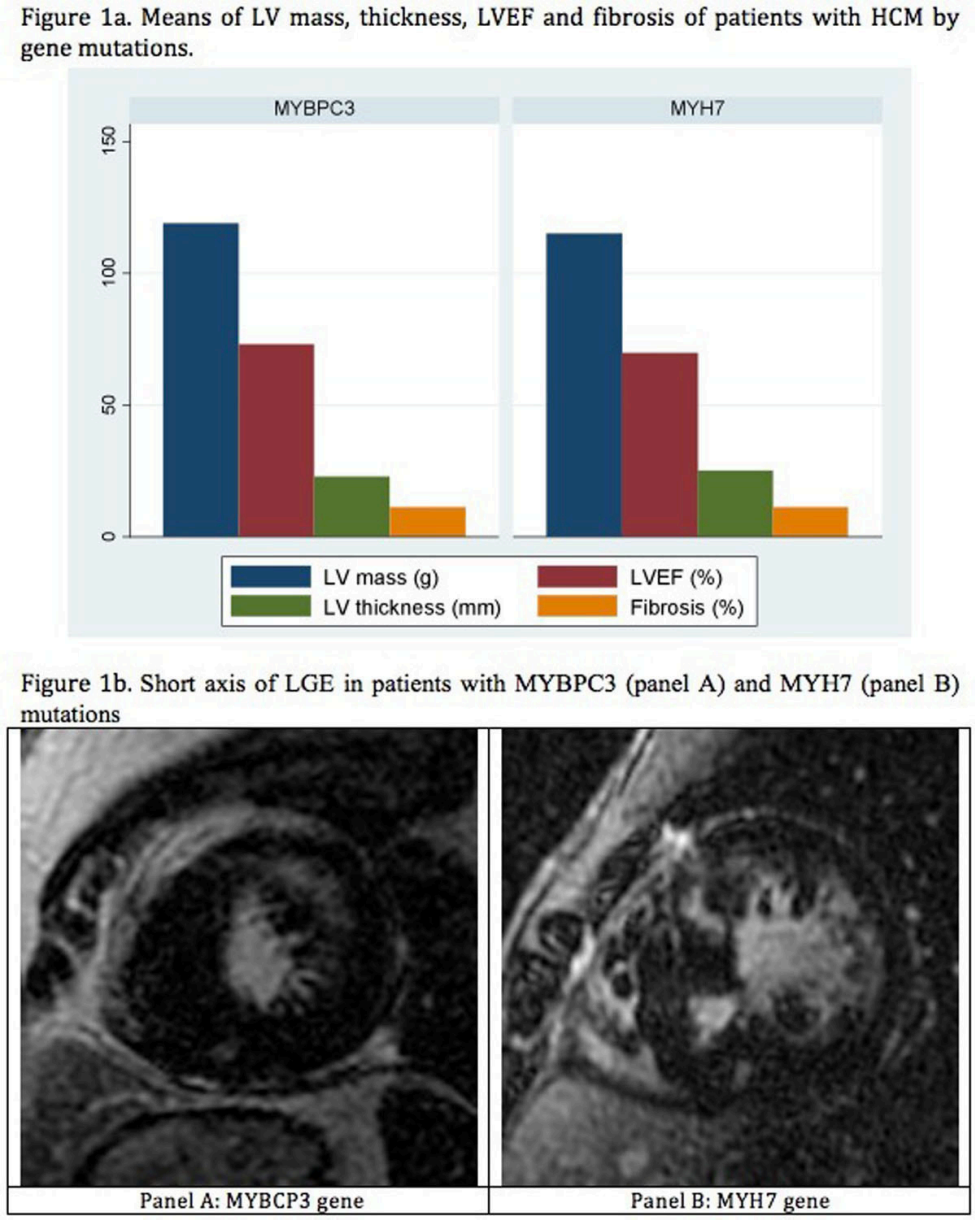# Myocardial fibrosis comparison by cmr between genetically positive HCM patients with MYBPC3 and MYH7 gene mutations

**DOI:** 10.1186/1532-429X-17-S1-P348

**Published:** 2015-02-03

**Authors:** Alejandra Villanueva, Liliane Rocha, Antonildes N Assunção Jr, Gabriela Liberato, Maria Solange A Sanchez, Bernardo B Lopes, Jose E Krieger, Julia D Marsiglia, Alexandre Pereira, Edmundo Arteaga, Roberto Kalil, Carlos E Rochitte

**Affiliations:** 1 Heart Institute, InCor, University of Sao Paulo Medical School, Sao Paulo, Brazil

## Background

Advances in tissue characterization with late gadolinium enhancement (LGE) by cardiovascular magnetic resonance (CMR) have highlighted the importance of myocardial fibrosis (MF) in hypertrophic cardiomyopathy (HCM) by confirming that its presence and extent predicts adverse outcomes. Despite of the identification of several genes related to HCM, few studies have investigated the association between genotype and MF. In this study, we sought to investigate the relationship between two most common gene mutations in HCM and the extension of MF by LGE.

## Methods

We retrospectively analyzed 57 patients with HCM and genetically positive for MYBPC3 or MYH7. All patients had CMR examination at 1.5 T MRI system (Philips Achieva). All patients underwent cine-MR with SSFP sequence for left ventricle function evaluation and late gadolinium enhancement for myocardial fibrosis detection. Myocardial fibrosis was measured by a thresholding technique above normal myocardium. Left vnetricle ejection fraction, mass and septum thickness were also calculated. All analyses were performed using CVi42 software (Circle CVi, Calgary, CA). HCM patients underwent clinical genetic testing on lymphocyte-derived DNA. Genes were sequenced through a standard Sanger sequencing protocol. Here we have only analyzed patients in which a causal mutation was identified in either MYBPC3 or MYH7.

Fisher exact test, t test and Mann Whitney test when appropriate using Stata 12.

## Results

The MYBPC3 gene mutation was present in 24 patients (42.1%) and the MYH7 in 33 patients (57.8%). The majority of the patients was male in both subgroups, 66.6% and 63.4%, respectively, and the mean age was similar (36.5 *vs* 37.2). Myocardial dysfunction was rare in this study, with only two patients presenting LV dysfunction (40% and 45%). Other characteristics were similar between groups (Table 1). There was no difference in the MF extent between genetically positive HCM patients with MYBPC3 and MYH7 gene mutations (11.0% ± 2.39 vs 11.0 ± 1.44, p=0.38) (Figure 1a). Figure 1b shows two short axis of LGE of patients with MYBPC3 (panel A) and MYH7 (panel B) mutations.

**Table 1:** Clinical and CMR characteristics of patients with HCM and genetically positive for MYBPC3 or MYH7.

	All (n=57)	MYBPC3	MYH7	P-value
Age, years ± SD	36.8 ± 12.3	36.5 ± 12.2	37.2 ± 12.6	0.84

Male, n (%)	37 (64.9)	16 (66.6)	21 (63.4)	0.64

LVEF, % ± SD	71.1 ± 10.9	72.9 ± 8.5	69.6 ± 12.5	0.53

LV mass, g ± SD	207.5 ± 70.4	216.7 ± 72.9	202.2 ± 69.8	0.54

Thickness, mm ± SD	23.9 ± 6.9	22.7 ± 1.69	25.1 ± 1.24	0.24

Fibrosis (%)	11.0 ± 9.7	11.0 ± 2.39	11.0±1.44	0.38

Fibrosis, g ± SD	21.6 ± 21.0	21.4 ± 25.3	21.7 ± 17.8	0.47

## Conclusions

In our group of HCM patients, MYBPC3 and MYH7 gene mutations subgroups had similar phenotype regarding the extent of the myocardial fibrosis measured by late gadolinium enhancement CMR.

## Funding

N/A.

**Figure 1:**